# Soluble Alpha-APP (sAPPalpha) Regulates CDK5 Expression and Activity in Neurons

**DOI:** 10.1371/journal.pone.0065920

**Published:** 2013-06-11

**Authors:** Daniela Hartl, Stephan Klatt, Manfred Roch, Zoltan Konthur, Joachim Klose, Thomas E. Willnow, Michael Rohe

**Affiliations:** 1 Institute for Medical Genetics and Human Genetics, Charité - University Medicine, Berlin, Germany; 2 Max Planck Institute for Molecular Genetics, Berlin, Germany; 3 Free University Berlin, Faculty of Biology, Chemistry and Pharmacy; Berlin, Germany; 4 Max Planck Institute of Colloids and Interfaces, Potsdam, Germany; 5 Max-Delbrueck-Center for Molecular Medicine, Berlin, Germany; Oregon Health and Science University, United States of America

## Abstract

A growing body of evidence suggests a role for soluble alpha-amyloid precursor protein (sAPPalpha) in pathomechanisms of Alzheimer disease (AD). This cleavage product of APP was identified to have neurotrophic properties. However, it remained enigmatic what proteins, targeted by sAPPalpha, might be involved in such neuroprotective actions. Here, we used high-resolution two-dimensional polyacrylamide gel electrophoresis to analyze proteome changes downstream of sAPPalpha in neurons. We present evidence that sAPPalpha regulates expression and activity of CDK5, a kinase that plays an important role in AD pathology. We also identified the cytoprotective chaperone ORP150 to be induced by sAPPalpha as part of this protective response. Finally, we present functional evidence that the sAPPalpha receptor SORLA is essential to mediate such molecular functions of sAPPalpha in neurons.

## Introduction

Amyloid precursor protein (APP) is a major etiologic agent in Alzheimer disease (AD). Amyloidogenic processing of APP by beta- and gamma-secretases generates Amyloid-beta (Abeta), the main component of senile plaques. Accumulating evidence supports the notion that progression of AD is correlated with progressive accumulation of Abeta in the brain which can be caused by elevated production and aggregation or impaired clearance of the peptide [1]. More recently, a growing body of evidence also implicated another APP cleavage product, soluble alpha-APP (sAPPalpha), in AD pathology. This product is produced from APP by an alternative, non-amyloidogenic processing pathway and levels of sAPPalpha were shown to be reduced in the cerebrospinal fluid of humans with sporadic or familial AD [2]–[4].

Important evidence for a central function of sAPPalpha in the brain was provided by studies with mice deficient for APP and APLP2, an APP homolog with overlapping functions. Perinatal lethality as well as other phenotypical abnormalities of APP/APLP2 double knockout mice were rescued by a sAPPalpha transgene [5]. Furthermore, if infused into the brains of rodents, sAPPalpha enhanced synaptogenesis and memory formation [6], [7]. Other studies reported enhanced survival of cells and neurite outgrowth after application of sAPPalpha to cultured cortical and hippocampal neurons [8]–[11] and other cell types [12]–[14]. Together, sAPPalpha was proposed to have neurotrophic and neuroprotective properties, possibly counteracting neurotoxic effects of Abeta. Accordingly, loss of sAPPalpha as observed in AD patients might contribute to disease pathology.

After cleavage of APP by alpha-secretase, sAPPalpha is released into the extracellular space in a process that was reported to be coupled to synaptic activity [15]. Only little is known about mechanisms controlling receptor-mediated uptake and downstream signalling of sAPPalpha in neurons. However, recent evidence suggested that sorting protein-related receptor containing LDLR class A repeats (SORLA; also known as LR11), an important AD risk factor, may act as sAPPalpha receptor [16], [17].

Here, we have used high-resolution two-dimensional polyacrylamide gel electrophoresis to determine proteins altered in expression after sAPPalpha application to primary cortical mouse neurons. We show that sAPPalpha regulates expression and activity of cyclin-dependent kinase 5 (CDK5), a kinase that plays an important role in AD pathology and that was previously shown to be activated by Abeta. We also identified hypoxia up-regulated protein 1 (ORP150) as effector protein potentially mediating neuroprotective functions of sAPPalpha. Finally, we present functional evidence that the sAPPalpha receptor SORLA determines these molecular functions of sAPPalpha.

## Materials and Methods

### Ethics Statement

All experiments performed with mice were conducted according to the guidelines of the German Animal Welfare Law. The study was approved by the State Office of Health and Social Affairs Berlin (approval number T0297/01).

### Preparation and Treatment of Primary Neurons

Primary cortical neurons were prepared from newborn Balb/c mice of either sex at postnatal day 1. Cortices were dissociated in papain (1 hour at 37°C) and cultured on poly-D-lysine/collagen coated culture dishes. The neurons were cultured for 4–5 days in Neurobasal-A medium (Gibco) including B27 supplement (Sigma), and GlutaMAX (Invitrogen) as previously described [18].

Neurons were treated with human recombinant neuron-specific sAPPalpha (APP isoform 695) produced in *E. coli* (SIGMA; for proteome analysis) or *Leishmania tarentolae* (for Western blot analysis) prepared as described before [19]. Neurons were supplied with sAPPalpha (300 ng/ml) or medium only (control) for one hour or 48 hours, respectively, by replacing half of the culture medium with fresh medium. For proteome analyses, the cells were harvested in ice cold PBS and cell pellets were frozen immediately in liquid nitrogen. Six individual samples of each, treated and control cells were collected for each condition (n = 6). Generation of animals genetically deficient for *Sorl1^−/−^* has been described before [20].

For detection of sAPPalpha, human specific monoclonal antibody (clone 2B3, IBL Hamburg), which recognizes the C-terminus of human sAPPalpha (DAEFRHDSGYEVHHQK) was used. All other antisera have been obtained from Cell Signaling Technology. Western blotting was conducted according to standard procedures. Protein signals were measured with a CCD camera based chemiluminescence imaging system (Peqlab). Quantification of signal intensities was accomplished with ImageJ software and significance of changes was determined applying Mann-Whitney-U test (p≤0.05) using Prism (version 5.0c, GraphPad).

### Protein Extraction and 2-D Electrophoresis

For proteome analyses, the cells were harvested in ice cold PBS. Six individual samples of each treated and control cells were collected (n = 6). Protein extracts were prepared from frozen cell pellets as described [21]. Briefly, samples together with sample buffer (50 mM TRIZMA Base (Sigma-Aldrich), 50 mM KCl and 20% w/v glycerol at pH 7.5) as well as proteinase and phosphatase inhibitors (Complete and PhosStop, Roche Diagnostics) were ground to fine powder in liquid nitrogen and sonicated on ice subsequently. Afterwards, DNAse (Benzonase, Merck), urea and thiourea (Biorad; 6.5 M and 2 M, respectively) were added. Protein extracts were then supplied with 70 mM dithiothreitol (Biorad), 2% v/w of ampholyte mixture (Servalyte pH 2–4, Serva) and stored at −80°C until separation by 2-D electrophoresis.

High-resolution, large-gel 2D-electrophoresis was described previously [22]. The gel format was 40 cm (isoelectric focusing)×30 cm (SDS-PAGE)×0.9 mm (gel width). Proteins were first separated according to their isoelectric points (isoelectric focussing, IEF) using the carrier-ampholyte technique [21], [22]. 40 µg of protein was applied to the acidic end of IEF gels (40 cm) and a carrier ampholyte mixture was added to establish a pH gradient spanning a range from pH 3 to 10. For SDS-PAGE, IEF gels were cut in half and run as “acidic” and “basic” sides. Two-dimensional protein patterns were obtained by sliver staining of gels as described [22]. The 2-D images were scanned at 300 dpi and 16-bit gray scale and saved in Tiff format to avoid loss of quality due to compression.

Protein spot patterns were evaluated using Delta2D imaging software (version 4.0, Decodon). Percent volume of spot pixel intensities was used for quantitative analysis of protein expression by Delta2D as described before [21]. Paired Student’s t-test was applied to determine statistical significance of alterations (significance threshold p≤0.05) as described before [21], [23]. Only fold changes exceeding 20% were considered.

### Mass Spectrometry

For protein identification, 1200 µg protein extract each was separated on a 2-D gel and stained with a MS-compatible silver staining protocol [22]. Protein spots of interest were excised from gels and subjected to in-gel tryptic digestion followed by HPLC separation as described [22]. Peptides were characterized by an ESI-tandem-MS/MS on a LCQ Deca XP ion trap instrument (Thermo Finnigan, Waltham, MA). Mass spectra were evaluated using MASCOT (version 2.1) automatically searching SwissProt database (version 51.8/513877 sequences). MS/MS ion search was performed with the following set of parameters: (i) taxonomy: Mus musculus, (ii) proteolytic enzyme: trypsin, (iii) maximum of accepted missed cleavages: 1, (iv) mass value: monoisotopic, (v) peptide mass tolerance 0.8 Da, (vi) fragment mass tolerance: 0.8 Da, (vii) fixed modifications: none and (viii) variable modifications: oxidation of methionine and acrylamide adducts (propionamide) on cysteine. Only proteins with scores corresponding to p<0.05, with at least two independent peptides identified were considered. The cut-off score for individual peptides was equivalent to p<0.05 for each peptide as calculated by MASCOT.

## Results

### Proteome Analysis of Murine Neurons Treated with sAPPalpha

Cumulative evidence suggests neurotrophic properties of sAPPalpha [6]–[11], [24], [25]. However, little is known about the underlying molecular mechanisms. We performed high-resolution 2-D electrophoresis (2-DE) combined with mass spectrometry to screen for proteins altered downstream of sAPPalpha signalling in neurons. This 2-DE based approach allowed us to simultaneously quantify expression levels of several modified forms of the same protein as these appear as separate spots on 2-D gels allowing separate quantification. This aspect deemed particularly important as neurotrophic factors often induce changes in post-translational modification of target proteins such as phosphorylation [19], [26].

Primary cortical neurons of mice were treated with sAPPalpha and proteome alterations following this treatment were determined by statistic evaluation of protein intensity signals on silver-stained 2-D gels using Delta2D software. In detail, cortical neurons were prepared from newborn mice and differentiated in culture for 4 days. Six individual preparations of primary neurons were compared in every group (n = 6, biological replicates) and every treatment condition was compared to controls (neurons treated with medium only). Recombinant sAPPalpha was applied to the cell culture media at a concentration of 300 ng/ml. The applied sAPPalpha concentration was described before to induce ERK phosphorylation in neurons [19] and to increase levels of neuroprotective genes when applied to organotypic hippocampal slice cultures [25].

**Figure 1:**
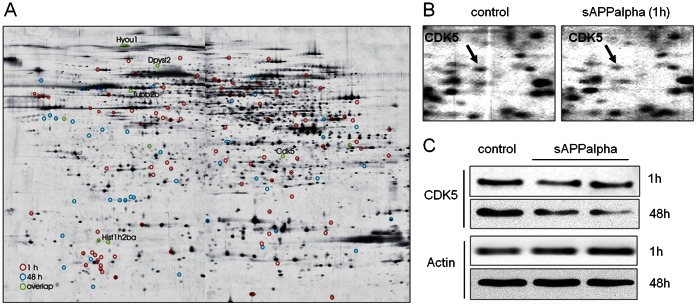
Protein expression changes in neurons treated with sAPPalpha. (**A**) Prototypic two-dimensional polyacrylamid gel of proteins extracted from primary cortical neurons stained with silver nitrate. Significant protein spot alterations in response to sAPPalpha treatment (300 ng/ml) for one hour (red circles), 48 hours (blue circles) or in both treatment conditions (green circles) are indicated (n = 6 biological replicates per genotype; paired Student’s t-test p<0.05). Gene names of identified proteins altered in both treatment conditions are indicated at respective positions. (**B**) Magnification of gel region containing cyclin-dependent kinase 5 (CDK5). CDK5 is indicated in the non-treated (control) and treated (sAPPalpha) condition. (**C**) Western blots of CDK5 in primary cortical neurons treated with sAPPalpha for one hour or 48 hours, respectively. Treated neurons (sAPPalpha) show reduced expression levels of CDK5 as compared to control neurons (control). Actin served as loading control.

We treated neurons for one hour and 48 hours, respectively, to cover acute as well as long-term effects. Comparison of treated versus control neurons revealed that 99 protein spots were significantly altered after one hour of sAPPalpha treatment and 47 protein spots were significantly altered after 48 hours of treatment. Nine proteins were altered under both treatment conditions (paired Student’s t-test, p≤0.05, ratio cut-off ≥20%; Figure 1A).

Together, proteome analysis revealed a large number of acute protein alterations after sAPPalpha treatment. A lower number of proteins were altered under chronic conditions.

### Reduced Expression of CDK5 and Altered Expression of CDK5 Associated Proteins in Neurons Treated with sAPPalpha

In order to identify significantly altered proteins, corresponding protein spots were excised from 2-D gels and identified by mass spectrometry (Table S1). We were most interested in proteins altered under both treatment conditions, as these proteins are most likely particularly relevant to sAPPalpha function. Among proteins altered under both treatment conditions, five proteins could be identified by mass spectrometry. These proteins were collapsin-response mediator protein 2 (CRMP2), histone H2B, hypoxia up-regulated protein 1 (ORP150), beta-tubulin and cyclin-dependent kinase 5 (CDK5). Expression of protein spots corresponding to CRMP2 and ORP150 was up-regulated and expression of spots corresponding to CDK5, histone H2B, and beta-tubulin was down-regulated in treated neurons.

Interestingly, CDK5 and CRMP2 were previously associated with AD pathology [27], [28]. Moreover, CRMP2, histone H2B, and beta-tubulin interact with CDK5 [27], [29], [30]. CDK5 plays a critical role in AD pathomechanisms as CDK5 phosphorylates APP, tau, and BACE1, affecting both hallmarks of AD, amyloid and tau pathology [31]. We therefore decided to analyze CDK5 as sAPPalpha target in more detail.

Using Western blot analysis of primary cortical neurons, we confirmed down-regulation of CDK5 expression after acute as well as long-term treatment with sAPPalpha (Figure 1 C). Moreover, 24% of proteins altered after sAPPalpha treatment (Table S1) are known CDK5 associated proteins (13 out of 55 non-redundant identified proteins; Table 1).

**Table 1:** CDK5 associated proteins significantly altered in expression after treatment of neurons with sAPPalpha (1 h, 48 h indicate duration of treatment; ↑ up-regulation, ↓ down-regulation, ratio treated/control).

Treatment condition	Direction of regulation	Gene name	Protein name	Comments
1 h, 48 h	↑ (1.34); ↑ (1.37; 1.64); ↑ (1.52)	Crmp1; Dpysl2; Dpysl4	Dihydropyrimidinase-related protein/Collapsin-response mediator protein	Collapsin-response mediator proteins (CRMPs) are involved in apoptosis/proliferation, cell migration, and differentiation. CRMP2 binds to microtubules and regulates axon outgrowth in neurons. This action is regulated by phosphorylation (via CDK5- and other kinases) at sites hyperphosphorylated in Alzheimer disease [29], [35]. CRMPs are altered in expression after treatment of neurons with CDK5 inhibitor [38].
1 h, 48 h	↓ (0.52; 0.6; 0.61); ↑,↓ (0.83; 1.38)	Hist1h2ba; Hist1h4a	Histone H2B type 1-A; Histone H4	CDK5 phosphorylates a component of the histone deacetylase complex and thus regulates histone acetylation i.e. during neuronal cell death. CDK5 can also directly phosphorylates histones [31], [51].
48 h	↓ (0.8)	Stxbp1	Syntaxin-binding protein 1 (Munc18-1)	CDK5 promotes Munc18-1 phosphorylation and calcium-dependent exocytosis [52].
48 h	↑ (1.23; 1.35)	Cbx3	Chromobox protein homolog 3	Also known as HP1gamma; repressor of E2F-dependent transcription which is regulated by CDK5 in the nucleus [53], [54].
1 h, 48 h	↓ (0.71); ↓ (0.79; 0.81; 0.59)	Tubb2a; Tubb2b	Tubulin beta-2A chain;Tubulin beta-2B chain	CDK5 phosphorylates several tubulin associated proteins regulating tubulin dynamics [32]. CDK5 inhibition alters tubulin expression in neurons [39].
1 h	↑ (1.32); ↓ (0.69); ↑ (1.31)	Ina; Myh10; Npm1	Alpha-Internexin; Myosin-10; Nucleophosmin	CDK5 phosphorylation targets [55,56]. The intermediate-filament protein alpha-internexin was altered in neurons after CDK5 inhibition [38].

Together, identification of altered proteins revealed that CDK5 might be the key downstream mediator of sAPPalpha signaling in neurons.

### CDK5 as well as CDK5 Associated Proteins are not Regulated by sAPPalpha in Neurons Lacking SORLA

SORLA is documented to be an intracellular trafficking receptor regulating APP processing [32]. SORLA directly binds to APP within the d6 domain, which is also an integral part of sAPPalpha [17]. Recent evidence demonstrated that SORLA also binds and mediates internalization of sAPPalpha in neurons [16]. As SORLA is the best-known receptor for sAPPalpha in neurons, we analyzed whether the observed molecular effects of sAPPalpha were dependent on this receptor.

**Figure 2:**
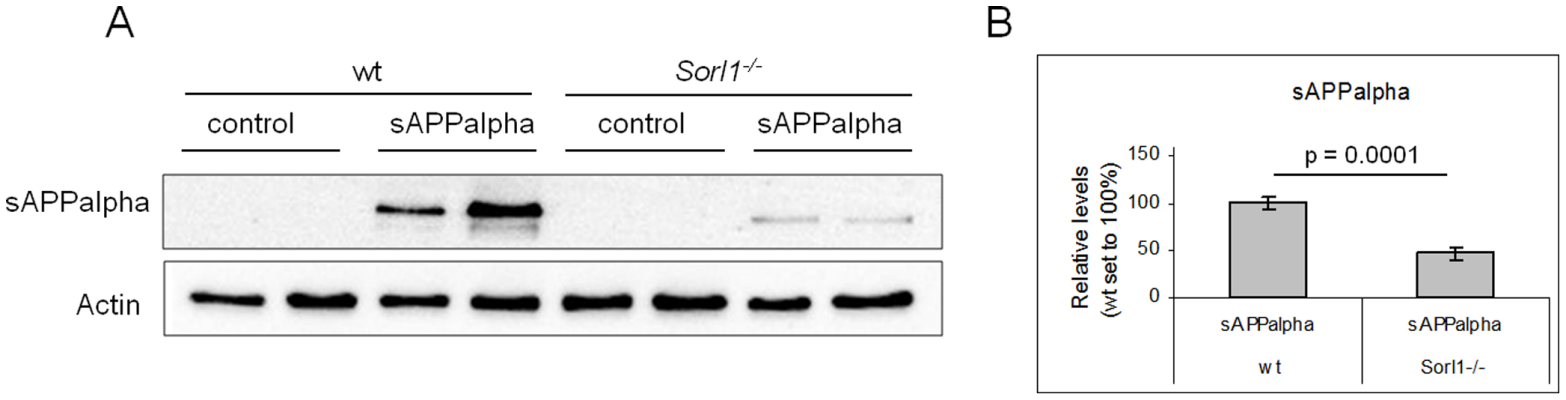
Reduced uptake of sAPPalpha in SORLA-deficient neurons. Quantification of human, recombinant sAPPalpha in primary cortical neurons either non-treated (control) or treated with 300 ng/ml sAPPalpha for 1 h using Western blotting (**A**) and densitometric scanning of replicate blots (**B**). *Sorl1^−/−^* neurons show reduced levels sAPPalpha after one hour of treatment compared with wild-type cells (wt; n = 8, Mann-Whitney U test). Actin served as loading control.

First, we analyzed whether SORLA mediates uptake of recombinant sAPPalpha in our experimental setup. Thus, we treated primary cortical neurons derived from either wild-type or *Sorl1* (the gene coding for SORLA)-deficient mice with sAPPalpha for one hour and quantified the amounts of intracellular recombinant sAPPalpha after treatment. In accordance with the previous study, we found a significant reduction of sAPPalpha signal intensity (about 50%) in *Sorl1*-deficient neurons as compared to wild-type neurons (Figure 2 A & B). This reduction is most likely due to reduced sAPPalpha uptake but it might also be the result of enhanced degradation.

**Figure 3:**
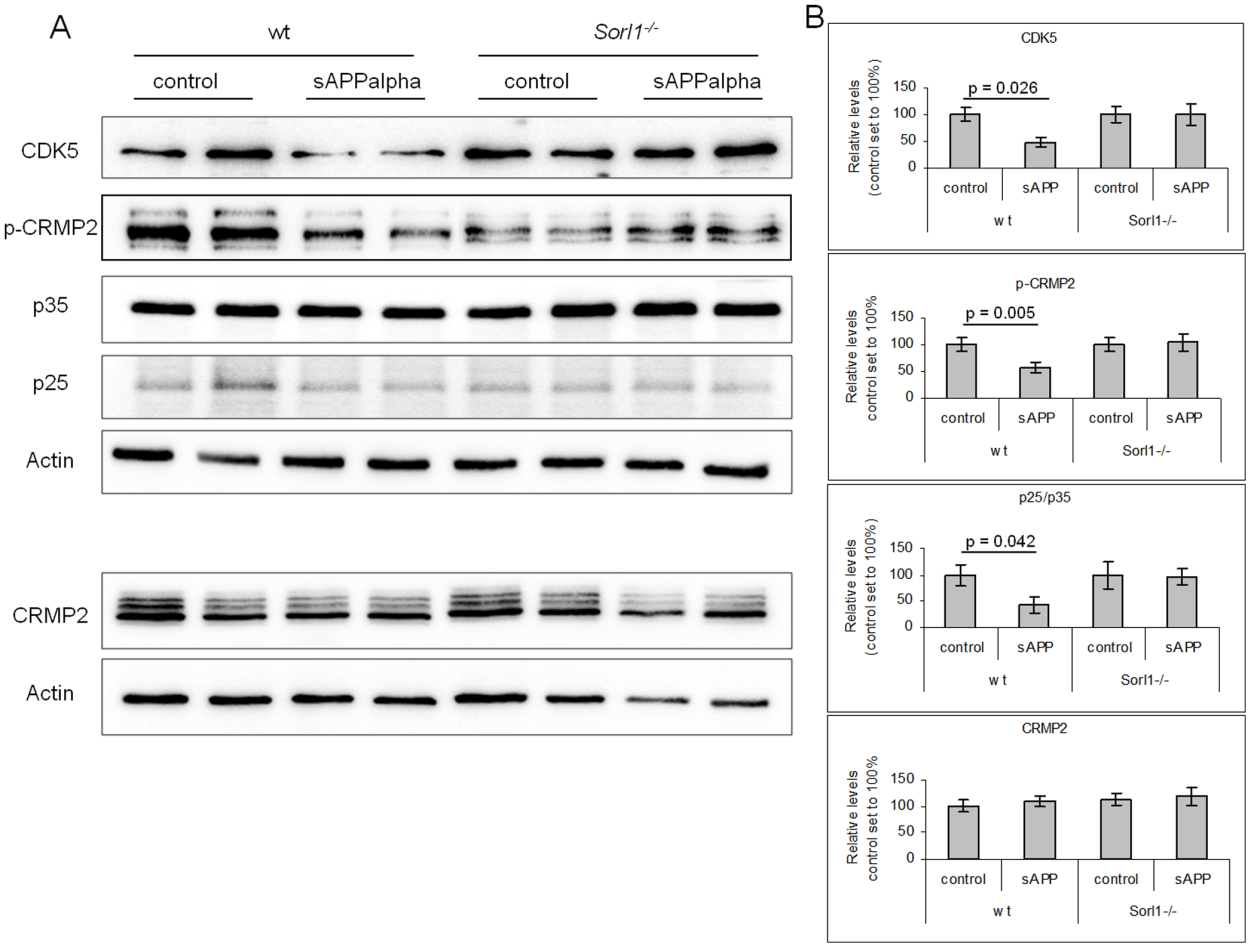
Reduction of CDK5 activity in neurons treated with sAPPalpha is SORLA-dependent. Quantification of CDK5, CDK5-target phospho-CRMP2, total CRMP2, and CDK5-adaptor proteins p35 and p25 in primary cortical neurons either non-treated (control) or treated with 300 ng/ml sAPPalpha for 1 h using Western blotting (**A**) and densitometric scanning of replicate blots (**B**). In wild- type neurons (wt), CDK5, p25 and phospho-CRMP2 (p-CRMP2) were significantly down-regulated after sAPPalpha treatment. *Sorl1^−/−^* neurons show no altered levels of the same proteins after one hour of sAPPalpha treatment (n = 6, Mann-Whitney U test). Actin served as loading control.

We next asked, whether regulation of CDK5 expression by sAPPalpha was also influenced by SORLA and if other proteins associated with CDK5 function were altered after sAPPalpha treatment. Western blot analysis of primary cortical neurons treated with sAPPalpha revealed that CDK5 and the CDK5 adaptor protein p25 (but not the CDK5 adaptor p35) were significantly down-regulated in wild-type neurons after treatment with sAPPalpha for one hour (Mann-Whitney-U test, p≤0.05, n = 6). As p25 is generated from p35 by calpain-dependent cleavage, we also quantified p25/p35 ratios. Also, the ratio of p25/p35 was significantly down-regulated (Figure 3 A & B).

One of the CDK5 target proteins identified in the proteome screen to be significantly regulated upon sAPPalpha treatment was collapsin-response mediator protein 2 (CRMP2). CRMP2 is phosphorylated by CDK5 at Ser522 [33]. Analyzing the phosphorylation state of CRMP2 upon sAPPalpha addition revealed reduced CDK5 activity as phosphorylation of CRMP2 at Ser522 was significantly reduced in treated neurons (Mann-Whitney-U test, p≤0.05, n = 6).

In contrast, CDK5 was not significantly altered in *Sorl1*-deficient neurons after sAPPalpha treatment (Figure 3 A & B). The same finding was seen for expression of the CDK5-target phospho-CRMP2 (Ser522) and the CDK5 adaptor proteins p35 and p25 (Mann-Whitney-U test, p≤0.05, n = 6, Figure 3 A & B). Reduction of phospho-CRMP2 (Ser522) was also observed in non-treated *Sorl1*-deficient as compared to non-treated control neurons. This could be due to the fact that sAPPalpha levels are per se enhanced in *Sorl1*-deficient neurons due to altered APP processing [34].

Together, our results revealed that the expression of CDK5 and CDK5 associated proteins was altered after sAPPalpha treatment in neurons. Reduced phosphorylation of CRMP documented impaired CDK5 activity after sAPPalpha application. None of these alterations were detected in *Sorl1*-deficient neurons proving that SORLA is an essential sAPPalpha receptor.

### Induction of Neuroprotective ORP150 by sAPPalpha

Up-regulation of ORP150 was previously shown to protect neurons from hypoxia and excitotoxicity [35], [36]. Interestingly, expression of ORP150 was induced by sAPPalpha treatment under both treatment conditions (1 hour and 48 hours of treatment; Table S1). ORP150 might therefore be involved in sAPPalpha-mediated neuroprotection.

**Figure 4:**
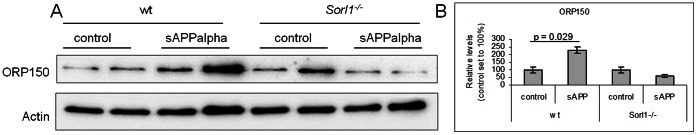
Induction of ORP150 in sAPPalpha treated neurons. Quantification of ORP150 in primary cortical neurons either non-treated (control) or treated with 300 ng/ml sAPPalpha for 1 h using Western blotting (**A**) and densitometric scanning of replicate blots (**B**). In wild-type neurons (wt), ORP150 is significantly up-regulated after sAPPalpha treatment. No alteration in ORP150 expression was observed in *Sorl1^−/−^* neurons after one hour of treatment (n = 6, Mann-Whitney U test). Actin served as loading control.

Western blot analysis of neurons treated with sAPPalpha confirmed significant up-regulation of ORP150 after sAPPalpha treatment of neurons. Again, this effect was not observed in neurons lacking the sAPPalpha receptor SORLA (Mann-Whitney-U test, p≤0.05, n = 6; Figure 4 A & B). Together, ORP150, a potential effector protein of sAPPalpha in neurons, was induced after sAPPalpha treatment. This effect was SORLA-dependent.

## Discussion

The APP cleavage product sAPPalpha is considered to be neurotrophic and neuroprotective [6]–[11], [24], [25]. Thus, loss of sAPPalpha activity in AD patients might contribute to disease pathology. However, little is known about molecular pathways underlying these effects. Using a 2-DE based proteomic approach, we uncovered that sAPPalpha significantly reduces expression and activity of CDK5 and influences expression of CDK5 target proteins in neurons. Interestingly, many of these CDK5 target proteins (cofilin, beta-tubulin, alpha-internexin and CRMP; table 1) were shown before to be altered in neurons treated with CDK5 inhibitors [37], [38]. This finding further supports a role of sAPPalpha in regulating CDK5 signaling.

CDK5 is an atypical cyclin kinase as -in contrast to other cyclin kinases- it inhibits the cell cycle to keep neurons in their post-mitotic stage. Transfer of CDK5 out of the nucleus induces neurodegeneration [39]. CDK5 also regulates neuronal morphology via phosphorylation of central components of the cellular cytoskeleton, such as tubulin, and tau [40]. Because CDK5 phosphorylates tau, APP, and BACE1, a component of the beta-secretase, CDK5 is an important link between amyloid- and tau-pathology [31]. Regulation of CDK5 activity by sAPPalpha was suggested before, as overexpression of sAPPalpha inhibited glutamate-induced CDK5 activation in N2a cells [41]. In contrast to sAPPalpha, Abeta activates CDK5 (reviewed in [42]). Aberrant activation of CDK5 can lead to collapse of the synaptic cytoskeleton [43]. sAPPalpha potentially protects from this collapse, as it maintains synaptic integrity [44]. Together, regulation of CDK5 activity in neurons might be the most important determinant of how the two alternative APP cleavage products Abeta and sAPPalpha counteract each other.

We also identified expression changes of the CDK5 phosphorylation target CRMP2 in neurons treated with sAPPalpha. Interestingly, CRMP2 phosphorylation impairs neurite outgrowth, an effect that can be reversed by inhibition of CDK5 [45]. We observed reduced phosphorylation of CRMP2 at the CDK5-dependent phosphorylation site (Ser522) in neurons treated with sAPPalpha. We further identified down-regulation of p25 after sAPPalpha treatment of neurons. p25 is a CDK5 adaptor protein that accumulates in the brains of AD patients leading to abnormal CDK5 activation and hyperphosphorylation of CDK5 targets, such as tau and CRMP2 [27], [42]. Together, down-regulation of p25 and phospho-CRMP2 demonstrate reduced activity of CDK5 in sAPPalpha treated neurons and further support the important role of sAPPalpha as neuroprotective factor.

One downstream effector protein providing neuroprotection might be ORP150, a protein induced in neurons after sAPPalpha treatment. ORP150 is an endoplasmic reticulum associated chaperone. Interestingly, induction of ORP150 protein has been demonstrated before in cultured neurons under hypoxic and excitotoxic stress conditions and in the brains of patients who died of epileptic seizures. However, overexpression of ORP150 protected neurons from hypoxia-induced cell death and mediated higher resistance of mice towards cerebral ischemia demonstrating that ORP150 protected cells from excitotoxicity [35], [36]. Interestingly, sAPPalpha has been reported to protect neurons from excitotoxicity as well [41]. Based on our findings we now speculate that induction of ORP150 by sAPPalpha might mediate this effect.

Finally, we provide functional evidence concerning the importance of SORLA as sAPPalpha receptor mediating not only uptake of sAPPalpha, but also controlling regulation of downstream sAPPalpha targets. Direct binding of SORLA to sAPPalpha and uptake of sAPPalpha by SORLA was shown before [16], [17]. We now document that SORLA is essential for the molecular function of sAPPalpha, as regulation of CDK5, phospho-CRMP2, p25 and ORP150 was no longer evident in neurons deficient for SORLA. As SORLA itself is considered an important genetic risk factor in AD [46] and low levels of this receptor have been documented in AD patients [47], it is likely that part of the neuroprotective properties ascribed to SORLA are related to its function as sAPPalpha receptor. Concerning its neuroprotective capabilities, so far SORLA was mainly recognized for its ability to regulate APP processing in the first place [20], [34], [48], but based in findings in this study SORLA might also serve as receptor of APP processing products controlling their respective cellular functions. That is at least the case for sAPPalpha.

### Conclusion

Our study provides insight into the molecular pathways downstream of sAPPalpha in neurons. We identified CDK5 and ORP150 as potential mediators of sAPPalpha-dependent neuroprotection. Moreover, we demonstrate a central role for SORLA as the sAPPalpha receptor; loss of sAPPalpha function in AD is a plausible mechanism of loss of sAPPalpha function in AD [44]. Here, we provide a proteomic-based evidence of how APP processing regulates neuronal function. We further propose that proteins regulated by sAPPalpha might be potential drug targets in their own right.

## Supporting Information

Table S1 Proteins identified as significantly altered in expression after 1 h or 48 h incubation with sAPPalpha. Proteins altered in both treatment conditions (1 h and 48 h) are highlighted in grey.
(PDF)
