# Supplementary material for: Soluble Alpha-APP (sAPPalpha) Regulates CDK5 Expression and Activity in Neurons
Source: PLoS One. 2013 Jun 11;8(6):e65920. doi: 10.1371/journal.pone.0065920 (PMC3679172; doi:10.1371/journal.pone.0065920)
Supplement: Table S1 — Proteins identified as significantly altered in expression after 1 h or 48 h incubation with sAPPalpha. Proteins altered in both treatment conditions (1 h and 48 h) are highlighted in grey. (PDF) [file pone.0065920.s001.pdf]

Supplementary table 1: Proteins identified as significantly altered in expression after 1 h or 48 h incubation with sAPPalpha. Proteins altered in both treatment conditions (1 h and 48 h) are highlighted in grey.

treatment condition	p-value	ratio (treated / control)	gene name	protein name	Unique peptides	Sequence coverage (%)
1h	0.048	1.240	Acaa1a	3-ketoacyl-CoA thiolase A, peroxisomal	8	19
1h	0.025	1.338	Aco2	Aconitate hydratase, mitochondrial	2	2
1h	0.025	1.391	Actb	Actin, cytoplasmic 1	6	22
1h	0.007	1.304	Actr1a	Alpha-centractin	6	18
1h	0.021	0.819	Arl3	ADP-ribosylation factor-like protein 3	3	19
1h	0.034	1.208	Atp5a1	ATP synthase subunit alpha, mitochondrial	20	33
1h	0.039	1.206	Atp5b	ATP synthase subunit beta, mitochondrial	19	31
1h	0.006	1.814	Atp5b	ATP synthase subunit beta, mitochondrial	4	13
1h	0.023	1.528	Atp6v1a	V-type proton ATPase catalytic subunit A	12	20
1h	0.012	0.779	Basp1	Brain acid soluble protein 1	8	58
48h	0.033	1.234	Cbx3	Chromobox protein homolog 3 (HP1-gamma)	2	7
48h	0.006	1.349	Cbx3	Chromobox protein homolog 3 (HP1-gamma)	2	7
1h	0.037	0.571	Cdk5	Cell division protein kinase 5	5	14
48h	0.050	0.751	Cdk5	Cell division protein kinase 5	5	14
48h	0.017	0.674	Cfl1	Cofilin-1	2	18
1h	0.004	1.537	Cops4	COP9 signalosome complex subunit 4	9	24
1h	0.010	1.369	Crmp1	Dihydropyrimidinase-related protein 1	2	5
1h	0.010	1.369	Dpysl2	Dihydropyrimidinase-related protein 2 (CRMP2)	2	5
48h	0.002	1.645	Dpysl2	Dihydropyrimidinase-related protein 2 (CRMP2)	5	12
1h	0.038	1.523	Dpysl4	Dihydropyrimidinase-related protein 4	2	5
1h	0.021	0.627	Eef1a1	Elongation factor Tu, mitochondrial	9	19
1h	0.042	2.060	Eef2	Elongation factor 2	9	10
1h	0.023	0.702	Endogl1	Nuclease EXOG, mitochondrial	4	10
1h	0.029	1.254	Erlin2	Erlin-2	9	22
1h	0.045	0.793	Fabp7	Fatty acid-binding protein, brain	3	31
48h	0.039	0.747	Fkbp2	FK506-binding protein 2	3	18
1h	0.039	1.328	Fscn1	Fascin	8	19
1h	0.033	0.828	Got2	Aspartate aminotransferase, mitochondrial	10	25
1h	0.035	0.525	Hist1h2ba	Histone H2B type 1-A	2	14
1h	0.005	0.598	Hist1h2ba	Histone H2B type 1-A	2	15
48h	0.026	0.613	Hist1h2ba	Histone H2B type 1-A	2	14
1h	0.043	0.826	Hist1h4a	Histone H4	2	19
1h	0.027	1.376	Hist1h4a	Histone H4	2	19
1h	0.023	1.643	Hmgcs1	Hydroxymethylglutaryl-CoA synthase, cytoplasmic	7	12
1h	0.036	0.825	Hnrnpa1	Heterogeneous nuclear ribonucleoprotein A1	8	29
1h	0.040	0.806	Hnrnpa1	Heterogeneous nuclear ribonucleoprotein A1	8	31
1h	0.023	0.721	Hnrnpa2b1	Heterogeneous nuclear ribonucleoproteins A2/B1	10	33
1h	0.008	1.250	Hsd17b8	Estradiol 17-beta-dehydrogenase 8	2	10
48h	0.025	1.440	Hyou1	Hypoxia up-regulated 1 (ORP150)	4	4
1h	0.043	1.246	Hyou1	Hypoxia up-regulated 1 (ORP150)	4	4
1h	0.011	1.253	Idh3a	Isocitrate dehydrogenase [NAD] subunit alpha, mitochondrial	10	29
1h	0.013	1.318	Ina	Alpha-Internexin	5	2
1h	0.007	0.775	Mtpn	Myotrophin	2	27
1h	0.046	0.693	Myh10	Myosin-10	3	2
1h	0.013	1.215	Ndufaf2	Mimitin, mitochondrial	8	51
1h	0.045	1.481	Ndufs3	NADH dehydrogenase [ubiquinone] iron-sulfur protein 3, mitochondrial	4	12
48h	0.016	0.801	Ndufs4	NADH dehydrogenase [ubiquinone] iron-sulfur protein 4, mitochondrial	4	25
1h	0.038	1.310	Npm1	Nucleophosmin	6	19
1h	0.002	1.263	Oxct1	Succinyl-CoA:3-ketoacid-coenzyme A transferase 1, mitochondrial	3	7
1h	0.050	0.816	Pcbp2	Poly(rC)-binding protein 2	2	8
1h	0.024	1.578	Pdcd6ip	Programmed cell death 6-interacting protein	2	3
1h	0.040	0.739	Pea15a	Astrocytic phosphoprotein PEA-15	2	22
1h	0.043	1.585	Ppia	Peptidyl-prolyl cis-trans isomerase A	5	37
1h	0.037	1.211	Psmd7	26S proteasome non-ATPase regulatory subunit 7	6	17
1h	0.045	0.821	Sh3bgrl	SH3 domain-binding glutamic acid-rich-like protein	5	44
1h	0.036	0.825	Slc25a22	Mitochondrial glutamate carrier 1	8	20
1h	0.029	1.692	Spr	Sepiapterin reductase	2	11
48h	0.048	0.803	Stxbp1	Syntaxin-binding protein 1	2	3
1h	0.022	0.787	Suclg1	Succinyl-CoA ligase [GDP-forming] subunit alpha, mitochondrial	3	11
1h	0.033	0.795	Tomm22	Mitochondrial import receptor subunit TOM22 homolog	2	8
1h	0.003	1.274	Trap1	Heat shock protein 75 kDa, mitochondrial	15	24
1h	0.048	0.708	Tubb2a	Tubulin beta-2A chain	2	6
1h	0.005	0.787	Tubb2b	Tubulin beta-2B chain	2	6
1h	0.025	0.818	Tubb2b	Tubulin beta-2B chain	5	12
48h	0.000	0.594	Tubb2b	Tubulin beta-2B chain	2	6
1h	0.028	0.731	Uqcrh	Cytochrome b-c1 complex subunit 6, mitochondrial	2	24
